# Host-Microbe Interactions: Understanding the Mechanism of Autophagy in Viral Replication and Immune Evasion

**DOI:** 10.3390/vetsci12121200

**Published:** 2025-12-15

**Authors:** Ziyuan Fu, Xiaowen Li, A. M. Abd El-Aty, Ridvan Yagan, Xianghong Ju, Yanhong Yong

**Affiliations:** 1 Marine Medical Research and Development Centre, Shenzhen Institute of Guangdong Ocean University, Shenzhen 518120, China; 2 College of Coastal Agricultural Sciences, Guangdong Ocean University, Zhanjiang 524088, China; 3 Zhanjiang City Key Laboratory of Animal Disease Prevention and Control Technology & Preparation Development, Shenzhen 518120, China; 4 Department of Pharmacology, Faculty of Veterinary Medicine, Cairo University, Giza 12211, Egypt; abdelaty44@hotmail.com; 5 Department of Medical Pharmacology, Medical Faculty, Ataturk University, Erzurum 25240, Turkey; 6 Department of Anatomy, Medical Faculty, Ataturk University, Erzurum 25240, Turkey

**Keywords:** autophagy, viral infection, immune regulation, selective autophagy, antiviral therapy, viral escape

## Abstract

Autophagy is a highly conserved catabolic process in eukaryotic cells that maintains cellular homeostasis by degrading damaged or superfluous intracellular components. Autophagy plays a dual, paradoxical role during viral infection. However, for most viruses, the induction of autophagy provides a favorable intracellular environment for the full completion of their life cycles. Most viruses that benefit from autophagy adopt a “regulate but not destroy” strategy, i.e., they initiate the autophagic process while suppressing their immune system through mechanisms such as blocking autophagosome-lysosome fusion. This allows them to avoid self-elimination while redirecting other functions of the autophagic machinery—for instance, utilizing autophagy-derived structures such as autophagosomes and double-membrane vesicles (DMVs) as specialized sites for viral genome replication, particle assembly, and maturation. The maintenance of cellular homeostasis by autophagy is crucial for the establishment of viral infection, as it provides a viable cellular microenvironment for viral replication; after infection occurs, inhibiting the degradative function of autophagy becomes a key strategy for viruses. Although canonical degradative autophagy exerts a negative effect on most viruses, redirected nondegradative autophagic structures and repurposed autophagic mechanisms are essential for the efficient replication of various viruses. In-depth analysis of this dynamic virus-autophagy interplay will provide important insights for elucidating virus-host interactions and developing autophagy-targeted antiviral strategies.

## Simple Summary

Understanding the mechanisms underlying virus–host interactions is crucial for advancing the development of prevention and control strategies for viral infectious diseases. This review focuses on the dual role of autophagy in viral infection and immune evasion. The multistep process of autophagy, encompassing initiation, nucleation, elongation, and fusion, is orchestrated by complex regulatory networks, such as the mTORC1/AMPK signaling pathway, the PI3K-Akt signaling pathway, p53, and epigenetic modulators. We then elaborate on how viruses hijack the autophagic machinery to facilitate their own lifecycle—from cellular entry and replication to assembly/release—and to evade host immune surveillance. Finally, we discuss the therapeutic potential of autophagy-targeting strategies for antiviral intervention, while acknowledging challenges such as cell type-specific functions and the emergence of viral mutations. Future research directions are proposed, focusing on deciphering the regulation of autophagy and the identification of precise molecular targets to optimize therapeutic efficacy.

## 1. Introduction

As obligate intracellular parasites, viruses rely entirely on the molecular machinery of host cells for their replication cycles. During long-term coevolution, hosts have evolved multilayered defense systems, among which autophagy is one of the highly conserved cell-autonomous defense mechanisms. Autophagy mediates degradation by forming double-membraned autophagosomes that sequester cytoplasmic components and fuse with lysosomes. This process not only maintains cellular homeostasis but also plays a pivotal role in orchestrating host immune responses to pathogen invasion [1]. Autophagy is of central importance in virology research, primarily because of its dual and paradoxical role in the viral life cycle: it serves as an innate immune effector for the host to clear invading viruses, yet its machinery is frequently hijacked by viruses, which repurpose it to facilitate viral entry, replication, and dissemination. This complexity makes elucidating virus-autophagy interactions essential for deciphering viral pathogenesis and developing innovative antiviral strategies. In recent years, with the progress of high-throughput sequencing and live-cell imaging technologies, the dynamic interplay between autophagy and viral infection has become increasingly clear—autophagy can act as the first line of innate immunity to directly clear viruses, or it can be subverted and exploited by viruses to promote their replication cycles [2,3].

From a public health perspective, understanding the interaction between autophagy and viruses holds significant clinical significance. Major pathogens, such as influenza A virus, severe acute respiratory syndrome coronavirus 2 (SARS-CoV-2), and human immunodeficiency virus (HIV), have demonstrated distinct and precise capacities to regulate autophagic mechanisms, and this regulatory relationship directly affects viral pathogenicity and the efficacy of host immune responses [4]. For example, SARS-CoV-2 blocks autolysosome formation through its ORF3a protein, leading to the intracellular accumulation of viral particles [5], whereas HIV indirectly inhibits the nuclear translocation of the transcription factor TFEB via the Nef protein, thereby downregulating autophagic activity to evade the host’s antiviral immune response to the virus [6]. These findings highlight the central role of autophagy regulation during viral infection and provide potential targets for the development of novel antiviral strategies.

This study will systematically review the molecular mechanisms underlying autophagy, analyze its dual functional modes during viral infection, focus on elaborating the strategies of viral autophagic escape and hijacking, and explore the potential of therapeutic intervention based on autophagy regulation, aiming to provide a comprehensive theoretical framework for basic research and translational applications in this field.

## 2. Molecular Mechanisms and Regulatory Networks of Autophagy

### 2.1. Core Molecular Mechanisms of Autophagy

**Figure 1:**
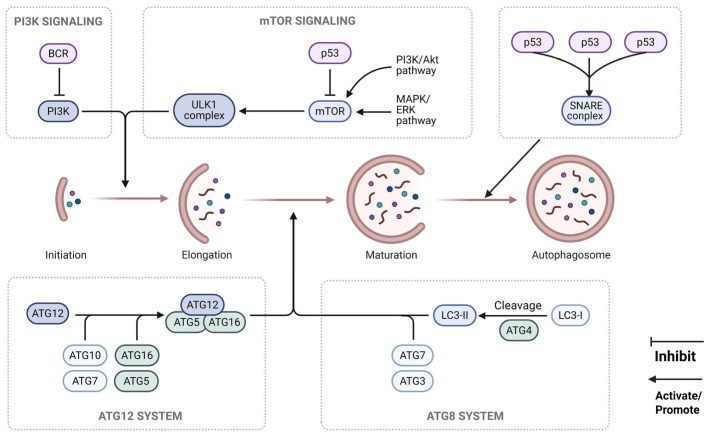
Autophagy-related regulatory mechanisms. This figure illustrates the activation of the intracellular autophagy mechanism and the signaling network that regulates autophagy. The autophagy process consists of four stages, namely, initiation, elongation, maturation, and autophagosome formation, which involve the synergistic action of multiple signaling pathways and protein systems. The initiation stage is driven by the PI3K signaling pathway: B-cell receptor (BCR) activates phosphatidylinositol 3-kinase (PI3K), which in turn regulates the downstream ULK1 complex to initiate the autophagy initiation process. ATG12 sequentially binds to ATG5 and ATG16 to form the ATG12-ATG5-ATG16 complex, which is involved in the elongation of the autophagic membrane. The maturation stage is subsequently mediated by the ATG8 system, and this stage is also regulated by the mTOR signaling pathway. Multiple p53 molecules regulate the SNARE complex to promote the final formation of autophagosomes; autophagosomes are double-membrane structures that enclose substrates to be degraded and subsequently fuse with lysosomes to complete the degradation and recycling of the substrates.

The autophagic process can be divided into four tightly coordinated stages: initiation, nucleation, elongation, and fusion (Figure 1, Table 1). The initiation stage is dominated by the ULK1 complex (comprising ULK1/2, Atg13, FIP200, and Atg101), whose activity is tightly regulated by cellular nutrient-sensing pathways [7]. Under nutrient-rich conditions, mTORC1 inhibits the activity of the ULK1 complex by phosphorylating ULK1 at the Ser757 site; under conditions of starvation or stress, mTORC1 is inactivated, and ULK1 undergoes autophosphorylation and phosphorylates Atg13 (as well as other downstream targets, such as AMBRA1 and mAtg9), triggering the autophagic program [8].

The nucleation stage depends on the activation of the autophagy-specific PI3K III complex (Complex I), which consists of Beclin1, Vps34 (class III PI3K), Vps15, ATG14 (Atg14L), and NRBF2. This complex recruits downstream effector proteins (e.g., WIPI1/2 and DFCP1) containing specific domains (e.g., FYVE and PX) to the autophagic initiation site by generating phosphatidylinositol-3-phosphate (PI3P) [9]. Like other members of the WIPI family, PI3P-binding proteins further recruit the Atg12-Atg5-Atg16L1 complex—a key mediator of membrane elongation, to promote the formation and expansion of the isolation membrane [10]. The activity of Beclin1 is regulated by various posttranslational modifications, including phosphorylation, ubiquitination, and acetylation, which orchestrate precisely control the occurrence of autophagy [26].

The extension of the autophagosome membrane requires the Atg12-Atg5-Atg16L1 complex to act as an E3-like enzyme to catalyze LC3 lipidation and thereby drives membrane elongation. Viruses such as hepatitis B virus (HBV) interact with Atg12 via their core proteins, hijacking this complex to serve as a scaffold for capsid assembly while evading autophagic degradation [11]. Atg12 is covalently conjugated to Atg5 via the E1-like enzyme Atg7 and the E2-like enzyme Atg10, forming the Atg12-Atg5 conjugate [12]. This conjugate further assembles with the Atg16L1 homodimer to form a heterotetrameric complex [(Atg12-Atg5)_2_-(Atg16L1)_2_] [13]. The complex binds to WIPI2b through the N-terminal domain of Atg16L1, and WIPI2b mediates its localization to the surface of the PI3P-enriched isolation membrane. Acting as an E3-like enzyme, this complex directly catalyzes the lipidation reaction between LC3-I and phosphatidylethanolamine (PE), generating membrane-anchored LC3-II [14]—a key step that drives the elongation of the autophagosomal membrane. The LC3 precursor is cleaved by Atg4 to generate LC3-I, which then binds to PE via the sequential action of Atg7 and Atg3 to form LC3-II, which is integrated into the autophagosomal membrane and serves as a canonical marker molecule for autophagosomes [15,16]. Additionally, studies have shown that the amphipathic α-helical (AH) domain at the C-terminus of Atg3 senses membrane curvature, promoting covalent binding between LC3-I and PE, thereby facilitating the directional expansion of the autophagosomal membrane [17].

The fusion of mature autophagosomes with lysosomes relies on the assembly of SNARE protein complexes (e.g., syntaxin17-VAMP8-SNAP29), which mediate the fusion of autophagosomes and lysosomes to achieve the degradation of sequestered substrates [18]. ATG14L, a subunit of the Beclin 1-PI3KC3 complex, simultaneously interacts with the SNARE complex STX17-SNAP29 as well as vesicle-associated membrane protein 8 (VAMP8). An adaptor protein called PLEKHM1 (a member of the pleckstrin homology domain-containing protein family) can simultaneously bind to LC3, RAB7, and STX17. PLEKHM1 facilitates the approximation of autophagosomes and lysosomes by recruiting the adaptor complex transmembrane ubiquitin-like domain protein 1 (HOPS, also known as TMUB1). Notably, at this fusion stage, ATG14L in the Beclin 1-VPS34 complex is replaced by UV resistance-associated gene protein (UVRAG) [19,20]. The efficiency of this process directly modulates autophagic flux, and various viruses achieve autophagic evasion precisely by disrupting this critical step.

### 2.2. Signaling Regulatory Networks of Autophagy

The regulation of autophagy is a highly integrated and sophisticated network system in which mTORC1 and AMPK serve as the core regulatory nodes. As a central nutrient-sensing hub, mTORC1 localizes to the lysosomal membrane via Rag GTPases under amino acid-sufficient conditions and inhibits autophagic initiation by phosphorylating ULK1 [21]. When cells experience energy shortage (increased AMP/ATP ratio), AMPK is activated, which promotes the activity of ULK1 by directly phosphorylating it at the Ser317 and Ser777 sites and simultaneously inhibits mTORC1 by phosphorylating TSC2, forming a dual activation mechanism autophagy initiation [22,23].

The PI3K-Akt pathway, a key transducer of growth factor signals, activates mTORC1 through the Akt-mediated phosphorylation of TSC2, thereby suppressing autophagy initiation [24]. This regulation is particularly important during viral infection, as many viruses create a cytoplasmic microenvironment conducive to replication by activating the PI3K–Akt–mTOR pathway. In addition, the tumor suppressor p53 exerts bidirectional regulation of autophagy: nuclear p53 promotes autophagy by transcriptionally activating autophagy-related genes such as DRAM1, whereas cytoplasmic p53 negatively regulates autophagy by inhibiting the activity of the ULK1 complex [25]. This subcellular localization-dependent regulatory pattern provides potential targets for viruses to manipulate autophagy.

Recent studies have uncovered the role of epigenetic regulation in autophagy; for example, the histone deacetylase LSD1 negatively regulates autophagy through the mTOR pathway, uncovering novel insights into the long-term modulation of autophagic activity [21]. These complex regulatory networks enable cells to precisely tailor their autophagic activity in response to nutrient status, energy levels, and stress signals, and viruses have evolved various strategies to target these regulatory nodes, thus optimizing their own replication and propagation within host cells 

## 3. Interaction Between Viruses and Autophagy

### 3.1. Autophagy Participates in the Host Antiviral Response

As a highly conserved catabolic process in eukaryotic cells, autophagy not only sustains cellular homeostasis but also serves as a critical component of the host innate immune defense system. Its role in the antiviral response is manifested primarily at multiple levels, including direct degradation of viral components, regulation of immune signaling pathways, and modulation of antigen presentation, conjointly form a key defensive barrier for the host against viral invasion.

First, autophagy can directly recognize, sequester, and degrade invading viral particles, viral proteins, or nucleic acids through the “xenophagy” mechanism, thereby restricting viral replication at the early stages of infection. For instance, for certain viral capsid proteins, host cell autophagy receptors (e.g., p62, NDP52, and TAX1BP1) can recognize ubiquitinated viral proteins and recruit them to nascent autophagosomes for ultimate degradation within autolysosomes. For example, during the replication of porcine epidemic diarrhea virus (PEDV), interferon-induced BST2 (bone marrow stromal cell antigen 2) can restrict viral release by tethering viral particles to the cell surface and can also inhibit viral replication by mediating autophagic degradation of the PEDV N protein through autophagy [27]. In infections with neurotropic viruses, CDKL5 can utilize the autophagy pathway to degrade viral components and clear viral proteins, thereby alleviating the accumulation of intracellular viral protein aggregates and promoting host cell survival [28].

Second, autophagy acts as a bidirectional regulator of immune homeostasis by precisely modulating the activity and stability of innate immune signaling pathways. On the one hand, autophagy can negatively regulate hyperactivated immune responses via feedback inhibition to prevent immunopathology. For example, autophagy mediates the degradation of key signaling molecules such as mitochondrial antiviral-signaling protein (MAVS) aggregates, preventing sustained overactivation of type I interferon response [29]. On the other hand, many viruses hijack this very “braking” mechanism to achieve immune evasion. They employ viral proteins as “molecular bridges” to recruit selective autophagy receptors (e.g., p62 and NBR1) to core innate immune signaling molecules such as MAVS, STING, and MDA5, mediating their autophagic degradation and thus suppressing interferon production at its source. For example, the PB1 proteins of influenza A virus (IAV) [30] and pseudorabies virus (PRV) [31] target MAVS for degradation by recruiting NBR1 and NDP52, respectively, effectively silencing the host’s antiviral alarm system.

Furthermore, autophagy plays a pivotal role in initiating adaptive immunity. By participating in the antigen processing and presentation process, autophagy helps process viral antigens and present them to MHC class I or II molecules, thereby activating virus-specific T-cell responses to eliminate infected cells.

In summary, autophagy is a multifunctional weapon antiviral weapon of the host. It can directly clear viral components such as a “cleaner” and finely tune the intensity of immune signals such as a “regulator.” However, the long-term “evolutionary arms race” between viruses and hosts has driven viruses to evolve diverse counterstrategies, manipulating the autophagy machinery to promote their own replication and evade immune clearance [1,4]. Therefore, an in-depth understanding of the dual role of autophagy in antiviral responses is crucial for developing novel therapeutic strategies that enhance host autophagic activity to combat viral infections.

### 3.2. Viral Utilization of Autophagy to Promote Self-Invasion and Genome Release

**Figure 2:**
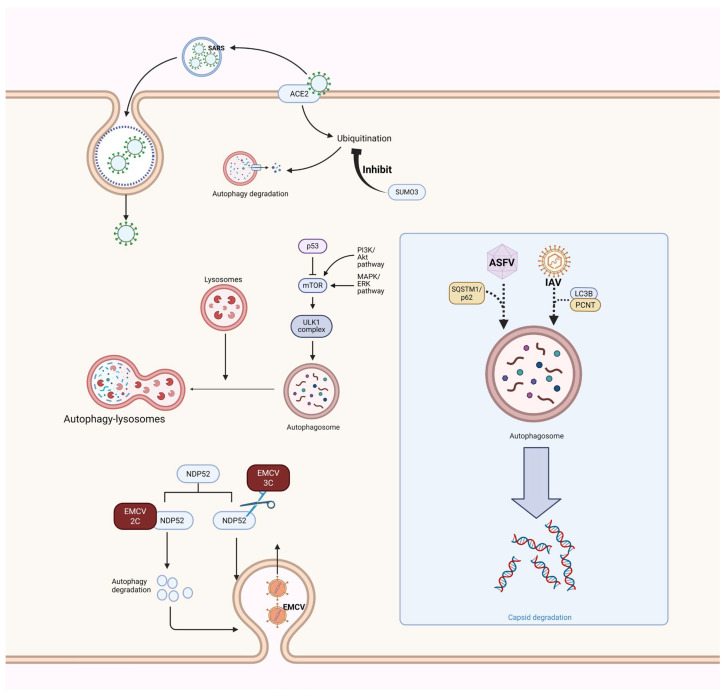
Viral Utilization of Autophagy to Mediate Self-Invasion and Genome Release. This figure presents the complex interaction between viruses and the autophagic system of host cells. The left side shows that viruses utilize the classic autophagic process to participate in their own internalization. SARS stabilizes the structure of ACE2 by inhibiting its autophagic degradation, thereby promoting its own invasion. The 2C and 3C proteins of EMCV affect NDP52 to facilitate infection; moreover, various viruses (e.g., ASFV, EBV, and DENV) utilize or regulate autophagosome structures after invading host cells and promote the degradation of capsid proteins through autophagosomes to release their own genomes (Created in Biorender. Ziyuan Fu. (2025) https://BioRender.com, accessed on 9 December 2025).

The viral life cycle can be divided into several core stages: attachment, entry, uncoating, replication, maturation, and release. Among these stages, viral attachment, entry, and uncoating are critical for the release of the viral genome (Figure 2, Table 2) [32]. During these early stages, various viruses harness the autophagy mechanism, which primarily facilitate viral infection in two ways: first, by regulating the stability of viral receptors, and second, by using autophagy-related membrane structures as platforms for invasion.

Coronaviruses are a classic example of receptor stability regulation via autophagy. Coronaviruses rely on their receptor (ACE2) to invade cells; thus, stable expression of ACE2 significantly influences the efficiency of their invasion. In severe acute respiratory SARS-CoV-2, its invasion receptor ACE2 interacts with the K187 site of the small ubiquitin-like modifier (SUMO3). This interaction inhibits the K48-linked ubiquitination of ACE2 itself, thereby suppressing TOLLIP-mediated autophagic degradation of ACE2. This enhances the stability of ACE2 and boosts viral infectivity [33]. However, after infection, the S protein of SARS-CoV-2 binds to ACE2 through its RBD and triggers cellular autophagy, leading to the degradation of ACE2. This process inhibits the innate immune mechanism of host cells and promotes the virus’s own replication [34]. During this process, the heat shock protein Hsp70 also interacts with the RBD of the SARS-CoV-2 S protein and ACE2, thereby modulating basal autophagic activity and contributing to SARS-CoV-2 [35].

Regarding the utilization of membrane platforms, autophagic membrane structures are directly co-opted by viruses for internalization or membrane fusion. For the highly lethal Ebola virus (EBOV), autophagy-derived membrane structures are critical for its cellular invasion. Previous studies have shown that after EBOV virions attach to the plasma membrane, the autophagic pathway is crucial for EBOV internalization. Autophagy-related molecules induce lysosomal degradation of protein aggregates and organelles during EBOV infection and interact with macropinocytic structures to promote viral internalization [36]. Recent research has identified PI3KC3 as a key molecule for EBOV invasion: PI3KC3 regulates autophagic membrane trafficking to enhance virion-cell binding, thereby facilitating viral invasion [34]. Similarly, autophagy induced by PI3KC3 affects the replication of other RNA viruses. For example, hepatitis C virus (HCV) interacts with PI3KC3 through its NS4B protein and targeting early endosomes to promote viral replication [37]. Autophagic membranes formed during autophagy also play important roles in the fusion process between viruses and cell membranes. Early during HIV-1 infection, LC3B rapidly accumulates on the target cell plasma membrane, which forms a complex with Atg8. This complex then undergoes lipid coupling, thereby promoting viral entry into cells [38].

In addition to promoting viral invasion, viruses can also harness autophagy to facilitate the disassembly of capsid proteins for genome release. During the invasion of African swine fever virus (ASFV), the E3 ubiquitin ligase Stub1 specifically recognize the K489 site at the C-terminus of the p72 protein and catalyzes the formation of polyubiquitin chains. The polyubiquitinated p72 is then degraded via p62-mediated autophagy, facilitating the release of the viral genome [39]. Studies of dengue virus (DENV) have also revealed that the autophagic cargo receptors p62, NDP52, and TAX1BP1 can interact with the viral C protein. Among these proteins, the UBA domain of p62 recognizes the K76-linked ubiquitination of the C protein and mediates its autophagic degradation, promoting the disassembly of the viral capsid [40]. Furthermore, following endocytosis and cellular entry, SARS-CoV-2 promotes the degradation of the ACE2-spike protein complex in an autophagy-dependent manner, thereby facilitating the release of its genome. Therefore, researchers have shown that inhibiting CE2 ubiquitination with drugs can attenuate the TOLLIP-mediated autophagic degradation of ACE2, thereby counteracting SARS-CoV-2 infection [33]. In addition, following endocytosis, IAV activates the autophagy-related molecule LC3B and promotes the formation of an adaptor complex between LC3B and pericentrin (PCNT), thus facilitating the release of its genome [41]. Moreover, numerous viruses induce cellular autophagy in the early stages of infection, but the role of this autophagy in viral early-life-cycle processes remains elusive. For example, autophagy occurs in cells 2 h post-infection with HCMV or herpes simplex virus type 1 (HSV-1), but its specific function has not been fully elucidated [42]. Therefore, elucidating the mechanism of autophagy in the early stages of viral infection is of great significance.

In summary, viruses facilitate their entry into host cells and the subsequent release of their genome through autophagy via multiple pathways, highlighting their sophisticated hijacking of host cellular mechanisms. This strategy is critical prerequisite for viruses’ successful invasion. It also opens up promising avenues for boosting the host’s antiviral defenses through targeted regulation of autophagic mechanisms—for instance, enhancing the autophagic degradation of viral receptors, or inhibiting autophagy to attenuate the degradation of viral capsids and the subsequent release of viral genomes.

**Table 2:** Viruses exploit autophagy to facilitate invasion and genome release.

Mechanism of Action	Virus	Hijacked Autophagy Steps/Molecules	References
Stable viral receptor	SARS-CoV-2	TOLLIP-mediated autophagic degradation pathway, Hsp70	[33,35]
Create an invasion membrane platform	EBOV	PI3KC3 complex, autophagic membrane trafficking	[34,36]
	HCV	Early endosome	[37]
	HIV-1	LC3B, Atg8	[38]
Create an invasion membrane platform	ASFV	p62-mediated selective autophagy	[39]
	DENV	p62, NDP52, TAX1BP1	[40]
	IAV	LC3B, pericentrin (PCNT)	[41]

### 3.3. Viral Utilization of Autophagy to Enhance Replication

**Figure 3:**
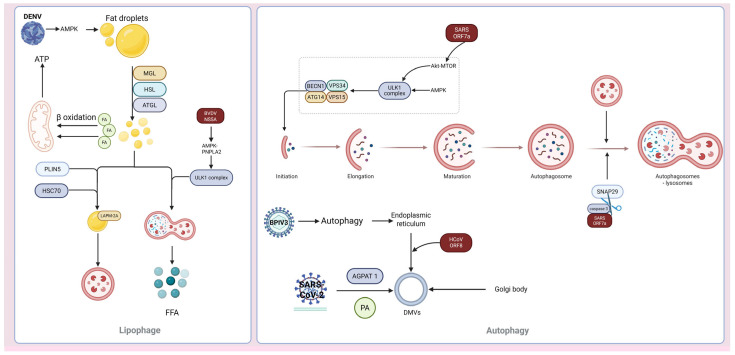
Viral Utilization of Autophagy to Modulate Self-Replication. This figure is divided into two panels, illustrating the complex interactions between viruses and host cell metabolism as well as the autophagic system. The left panel focuses on the associations between viruses and host lipid metabolism/autophagy. After activating AMPK, DENV influences fat droplet metabolism and participates in lipid decomposition. Moreover, proteins such as PLIN5 and ABHD5 regulate the interaction between lipid droplets and autophagosomes, ultimately forming “lipophages” that release free fatty acids (FFAs) to provide energy or substances for viral metabolism or replication. The NS5A protein of BVDV activates lipophagy by targeting AMPK-PNPLA2. The right panel presents the interactions between various viruses and the autophagic system: SARS ORF7a activates autophagy via the Akt-MTOR pathway and cleaves SNAP29 to promote its own replication. The ORF7a protein of SARS can also cleave SNAP29 to inhibit autophagic flux, forming autophagosome aggregates as replication factories. SARS, BPIV3, and HCoV can activate endoplasmic reticulum rearrangement through multiple pathways to form double-membrane vesicles (DMVs) (Created in Biorender. Ziyuan Fu. (2025) https://BioRender.com, accessed on 9 December 2025).

The replication of many viruses is associated with specific intracellular compartments termed viral factories or viroplasms (Figure 3, Table 3). Autophagosomes, which are generated during autophagy, can be hijacked by viruses to function as replication platforms [43]. Certain viruses inhibit the fusion of autophagosomes with lysosomes, leading to the accumulation of autophagosomes in cells; these accumulated autophagosomes are then hijacked to contribute to the assembly of viral replication factories. Furthermore, viruses can induce canonical autophagy and harness the autophagosomes the resulting autophagosomes to support viral replication. For example, classical swine fever virus induces complete autophagy in host cells and employs autophagosomes to assemble viral replication factories, which facilitate the production of the viral proteins NS5A and E2 [44].

**Figure 4:**
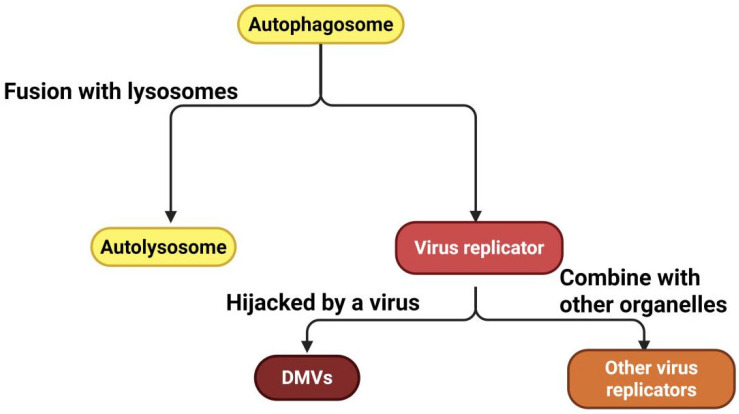
Relationships among autophagosomes, DMVs, and viral replicators. Viral replication organelles are functional microenvironments established by viruses within host cells for replication. Among these, double-membrane vesicles (DMVs) represent a typical form, primarily formed through the remodeling of host membrane systems (e.g., the endoplasmic reticulum) by viral proteins, often by hijacking components of the autophagy machinery. Other forms, such as lipid droplet-autophagosome complexes, are created through the association of different organelles. Together, these structures provide isolated compartments, essential building blocks, and energy to support viral replication (Created in Biorender. Ziyuan Fu. (2025) https://BioRender.com, accessed on 9 December 2025).

Recent studies have uncovered that “nondegradative autophagy” induced by some viruses is not simply a blockage of autophagic flux, but rather an active remodeling of the endomembrane system to generate double-membrane vesicles (DMVs) enriched with PI3P and LC3 (Figure 4). These structures are tightly linked to endoplasmic reticulum (ER)-mitochondria contact sites, providing a lipid-rich microenvironment and physical sequestration niche for viral RNA synthesis [45,46]. DMVs have multiple origins, including the ER and Golgi apparatus. Given that autophagosomes are surrounded by double membranes and many viruses have evolved mechanisms to hijack specific functions of autophagy-related factors to enhance their own replication, autophagosomes may also be involved in DMV biogenesis. For example, SARS-CoV-2 induces the formation of DMVs to create viral replication organelles [47]. In this process, the ORF8 protein of coronaviruses first interacts with the autophagic protein p62 to form phase-separated condensates and then binds to the ER-phagy receptors FAM134 and ATL3, hijacking these receptors into the ORF8/p62 phase separations. This suppresses ER-phagy, ensuring that the virus can remodel the ER to form DMVs, which facilitates viral replication [48]. In addition, phosphatidic acid (PA)—induced by the ER-localized acylglycerophosphate acyltransferases (AGPATs) 1 and 2, is not only involved in the formation of autophagic vesicles but also equally critical for DMV formation induced by HCV and SARS-CoV-2 [45]. Autophagy induced by endoplasmic reticulum stress seems to be a conserved strategy employed by viruses to promote their own replication. For example, bovine parainfluenza virus type 3 (BPIV3) induces ER stress by upregulating autophagy via GRP78, remodels the ER membrane, and thereby promotes viral replication [49]. Positive-sense RNA viruses form an ER-Golgi channel through the reticulophagy receptors ATL3 and RTN3L, mediating ER remodeling and vesicular trafficking and to support viral replication [50]. Therefore, in-depth exploration of the mechanism underlying ER rearrangement may reveal that ER rearrangement is a key replication factor for certain RNA viruses.

The modification of the host cell membrane system by viruses is not limited to conventional organelles such as the endoplasmic reticulum. In addition to remodeling the aforementioned internal membrane systems, viruses can further optimize their replication environment through another key strategy—namely targeting the cellular energy metabolism hub, lipid droplets. As highly dynamic organelles, lipid droplets maintain cellular lipid homeostasis through mechanisms including synthesis, breakdown, component fusion, and selective autophagy. Emerging evidence indicates that viruses can harness lipid droplet-autophagosome complexes as replication platforms. HCV and DENV employ lipid droplets to promote their replication. The newly synthesized RNA of HCV is transported to lipid droplets by the viral replicase proteins NS3 and NS5A; subsequently, the viral RNA, along with NS2 and P7 proteins, assembles into capsids within these organelles [51]. In recent years, it has been discovered that HCV not only uses lipid droplets as replication platforms but also induces lipophagy to modulate lipid droplet turnover, thereby supplying ATP for viral RNA replication, assembly, and trafficking [52,53]. DENV infection triggers lipophagy through either AMPK-dependent or AMPK-independent inhibition of mTORC1 activity. Through lipophagy, triglycerides in cellular lipid droplets undergo hydrolysis into free fatty acids, which are ultimately catabolized via β-oxidation to release ATP, thereby supporting efficient viral RNA replication [54]. Mechanistic studies have demonstrated that the tumor suppressor gene PTEN and its mutant Y138L enhance autophagic flux through the Akt/FoxO1/Maf1 signaling pathway, promote lipid droplet catabolism, and modulate lipid homeostasis to sustain the replication and assembly of DENV [55]. Research has revealed that the nonstructural protein NS5A of bovine viral diarrhea virus (BVDV) targets the AMPK-PNPLA2 pathway to induce lipophagy and liberate free fatty acids that support its own replication [56]. As another member of the Flaviviridae family, classical swine fever virus also employs lipid contribute to the assembly of viral replication factories [57].

In conclusion, viruses fulfill their requirements needs by inducing complete autophagy or incomplete autophagy or hijacking autophagy-related factors to contribute to the assembly of replication organelles. These findings offer promising targets for suppressing viral replication via targeted modulation of autophagy-related mechanisms, such as suppressing DMVs biogenesis and disrupting lipophagy.

**Table 3:** The virus uses autophagy to promote its replication.

Mechanism of Action	Virus	Hijacked Autophagy Steps/Molecules	References
Block autophagic flux and turn autophagosomes into replication factories	SARS-CoV-2	ORF7a protein, SNAP29, SNARE complex, p62, endoplasmic reticulum autophagy receptors (FAM134B, ATL3), Phosphatidic acid (PA), AGPAT1/2	[47,48]
	BPIV3	GRP78, ER stress	[49]
	HCV	Phosphatidic acid (PA), AGPAT1/2	[45]
Using the lipophagy complex	HCV	Lipid droplet, autophagosome	[51,52,53]
	DENV	AMPK signaling pathway, lipophagy	[54,55]
	BVDV	NS5A protein, AMPK-PNPLA2 pathway	[56]

### 3.4. Viral Utilization of Autophagy for Assembly and Release

**Figure 5:**
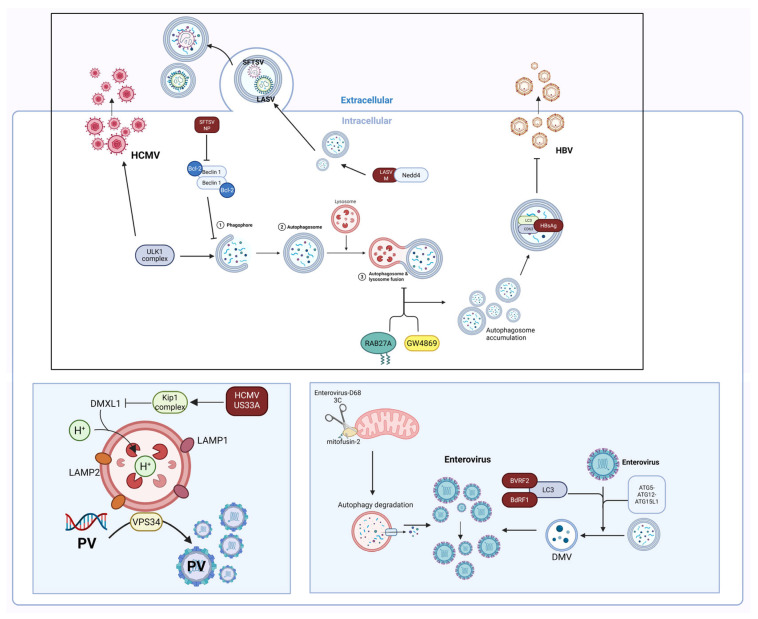
Viral Utilization of Autophagy to Enhance Self-Assembly and Release. This figure illustrates that viruses utilize autophagy through multiple pathways to participate in their own release. The NP protein of SFTSV inhibits the binding of BECN1 and BCL2, activates autophagy to form autophagosomes, and utilizes autophagosomes for extracellular release. HCMV and the poliovirus (PV) can utilize lysosomes and autophagosomes for assembly. Lyssavirus promotes viral budding through the interaction of its M protein with Nedd4. GW4869 (which inhibits ceramide-mediated inner membrane budding) can synergize with RAB27A to inhibit autophagic degradation, causing HBV to accumulate in autophagosomes and late endosomes/multivesicular bodies in the form of LC3-CD63-HBsAg complexes, thereby inhibiting viral secretion. EBV can interact with LC3 through the capsid scaffold proteins BVRF2 and BdRF1, induce the production of double-membrane vesicles (DMVs), and release these vesicles through fusion with the cell membrane. Enterovirus-D68 3C can cleave mitofusin-2 (a mitochondrial fusion protein), leading to mitochondrial fragmentation and mitophagic degradation, thereby promoting nonlytic release of the virus from cells (Created in Biorender. Ziyuan Fu. (2025) https://BioRender.com, accessed on 9 December 2025).

In the late stage of replication, viruses hijack the host autophagic system via multiple sophisticated strategies to accomplish the assembly and release of viral particles (Figure 5, Table 4). The core mechanisms encompass direct utilization of autophagosomes/lysosomes as assembly platforms, modification of autophagosomes to enable nondegradative release, selective employment of autophagic proteins as molecular scaffolds, and modulation of upstream autophagic signaling pathways. Enveloped viruses frequently utilize autophagosomes/lysosomes as either platforms or release carriers. Severe fever with thrombocytopenia syndrome virus (SFTSV) infection serves as a paradigm for viral utilization of autophagosomes as assembly platforms [58]. SFTSV induces autophagy (characterized by elevated LC3-II levels), and its NP protein interacts with the coiled-coil domain (CCD) of BECN1 to competitively inhibit BECN1-BCL2 binding, thereby activating autophagy and promoting autophagosome formation; SFTSV employs autophagosomes as assembly sites and subsequently releases virions into the extracellular milieu via an autophagosome-mediated exocytosis pathway [59]. The autophagy-initiating protein kinase ULK1 also exerts a positive regulatory role in the release of HCMV; inhibition of its upstream regulator AMPK indirectly disrupts ULK1 function and impair virion release [60]. The assembly of HCMV relies on lysosomal participation, with lysosomes detectable around the viral assembly compartment (vAC) formed by the virus [61]. HCMV recruits the Kip1 complex (KP3) E3 ligase via its US33A protein to mediate DMXL1 degradation (Figure 5), which suppresses lysosomal acidification and autophagic cargo degradation, thus delaying vAC formation [62]. Immunoelectron microscopy of infectious bursal disease virus (IBDV)-infected cells revealed numerous intact virions encapsulated within p62-surrounded vesicular membranes (with some p62 localized between virions), accompanied by a distinct release phase; inhibition of autophagosome-lysosome fusion or lysosomal hydrolytic activity impairs IBDV replication. These findings demonstrate that IBDV leverages the low-pH environment of acidic organelles to facilitate virion maturation and releases progeny viruses via a membrane-mediated pathway [63]. Following nervous system, lyssaviruses interact with the E3 ubiquitin ligase Nedd4 via the PPxY motif of their M protein, thereby inducing autophagosomes formation and further harnessing autophagosomes to promote viral budding [64]. This lysosome-independent autophagic release pathway enables allows lyssaviruses to evade the degradative lysosomal environment of lysosomes. As a γ-herpesvirus, Epstein–Barr virus (EBV) was initially shown utilize autophagy to mediate the fusion of the DMVs outer membrane with the cell membrane, facilitating viral release [65,66]. Subsequent studies further validated that this process is mediated by the interactions between the conserved regions of the viral capsid scaffold proteins BVRF2, BdRF1 and LC3 and requires the LC3-conjugating complex ATG5-ATG12-ATG16L1 [67]. The classical swine fever virus (CSFV) harnesses the autophagic pathway to generate extracellular vesicles, which shuttle mature virions from infected cells to neighboring uninfected cells, mediating intercellular transmission [68].

Nonenveloped viruses, incapable of being released via membrane fusion, have evolved distinct strategies to hijack the autophagy machinery. For example, hepatitis A virus (HAV) encapsulates its capsid within phosphatidylserine-depleted, LC3-positive “virus-like” membrane vesicles, and this release mode enables efficient evasion of neutralizing antibodies [69].

In addition to generating enveloped intact virions, HBV also produces and releases nonenveloped naked capsids during its secretion process (Figure 5) [70]. This process is independent of LC3 lipidation and autophagosome maturation; instead, it employs the Atg5-12/16L complex as a “molecular scaffold” for capsid assembly [56]. HBV facilitates the conjugation of Atg5-12 through catalysis by the E2-like enzymes Atg10 and Atg3; moreover, the core protein interacts with the partially disordered region of Atg12 and associates with the Atg5-12/16L1 complex, allowing Atg12 integration into virions and facilitating viral assembly [11]. Furthermore, a study on GW4869 (an inhibitor of ceramide-mediated endomembrane budding) demonstrated that GW4869 act synergistically with RAB27A to block autophagosome-lysosome fusion, enhance autophagosome formation and impair autophagic degradation. This leads to the accumulation of HBV HBsAg in autophagosomes and late endosomes/multivesicular bodies in the form of LC3-CD63-HBsAg complexes, thus suppressing viral secretion [71]. These results provide a model for investigating nonenveloped virus secretion. They hijack the upstream assembly scaffold of autophagy, abandon the downstream degradative function, utilize these complexes to promote capsid stabilization and assembly, and release progeny viruses into the extracellular milieu. On the basis of the above findings, it is reasonable to infer that hijacking specific components of the autophagic mechanism to achieve nonlytic release constitutes a “broad-spectrum strategy” convergently evolved by numerous nonenveloped viruses.

Notably, autophagy does not always facilitate viral release—it exerts the opposite effect during the herpes simplex virus type 1 (HSV-1). HSV-1-induced apoptosis requires caspase-8 [72], and caspase-8 knockout (caspase-8^−^/^−^) results in reduced virion release and enhanced autophagy (characterized by increased Beclin-1 levels, decreased p62/SQSTM1 levels, and enhanced conversion of LC3-I to LC3-II), resulting in the sequestration of virions within intracellular vesicles; however, the autophagy inhibitor wortmannin restores virions release [73]. These findings highlight that the autophagic system has become an indispensable “tool” in the viral life cycle. Investigating the interactions between viruses and autophagy-related molecules (e.g., ULK1 and Atg5-12/16L1) to develop strategies targeting viral assembly and release holds significant clinical value for antiviral therapy.

**Table 4:** Viruses use autophagy to promote assembly and release.

Mechanism of Action	Virus	Hijacked Autophagy Steps/Molecules	References
Using autophagosomes/lysosomes as assembly platforms and release carriers	SFTSV	NP protein, BECN1, autophagosome	[58,59]
	HCMV	US33A protein, DMXL1, lysosome, Kip1 complex	[60,61,62]
	IBDV	Autophagosome/lysosome, p62	[63]
	lysesaviruses	E3 ubiquitin ligase Nedd4, autophagosomes	[64]
Hijacking autophagy proteins as molecular scaffolds	HBV	Atg5-12-Atg16L1 complex	[11,56,70,71]
	EBV	BVRF2, BdRF1, LC3-conjugating complex ATG5-ATG12-ATG16L1	[67]
Using autophagosome membranes for disguised release	HAV	LC3-positive ‘virus-like’ membrane vesicles	[73]

### 3.5. Viral Regulation of Autophagy for Immune Evasion

**Figure 6:**
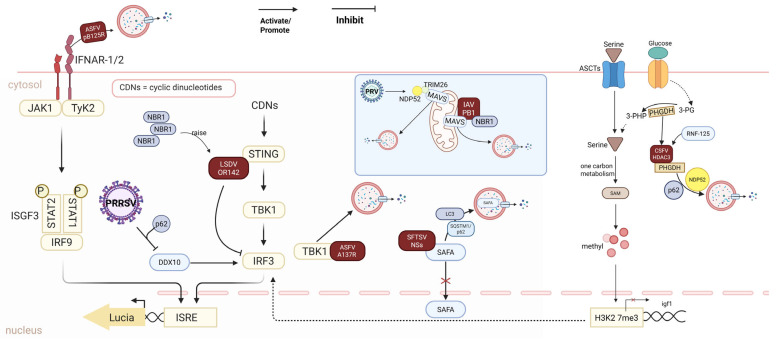
Viral immune evasion mechanisms. This figure illustrates various mechanisms by which viruses exploit autophagy to evade the host’s immune defense. ASFV pB125R escapes immunity by promoting the autophagic degradation of IFNAR-2. PRRSV inhibits the activation of IRF3 by DDX10 through p62-mediated autophagy. LSDV OR142 recruits NBR1 and directly interacts with STING to inhibit IRF3 activation. ASFV A137R interacts with TBK1 to promote its autophagic degradation. SFTSV NSs can sequester SAFA in the cytoplasm and promote its autophagic degradation, blocking SAFA-dependent antiviral enhancer activation. PRV and IAV can target MAVS for autophagic degradation. CSFV degrades PHGDH, a key enzyme in serine metabolism, through HDAC3, indirectly affecting mitochondrial function and inhibiting the MAVS-IRF3 pathway, which also involves the recruitment of p62 and NDP52 (Created in Biorender. Ziyuan Fu. (2025) https://BioRender.com, accessed on 9 December 2025).

Autophagy is not only a catabolic process that degrades cytoplasmic components but also a key regulator of innate immune signal transduction (Figure 6, Table 5). It directly participates in immune activation by degrading immune signaling molecules or viral nucleic acids; conversely, viruses have also evolved sophisticated strategies to hijack this “double-edged sword” function, particularly the selective autophagy pathway, to degrade core immune adaptor proteins and achieve immune evasion. This section systematically elaborates on how viruses utilize autophagy to regulate the type I interferon pathway.

The core mechanisms by which viruses achieve immune evasion via autophagy can be summarized as follows: 1. Targeting and recognizing pattern recognition receptors (PRRs) or adaptor proteins (e.g., MAVS and STING) and 2. Recruiting selective autophagy receptors (e.g., p62 and NBR1) to deliver these molecules to autolysosomes for degradation. For example, the Avian influenza A (H7N9) PB1 protein induces K27-linked ubiquitination of MAVS and recruits NBR1, thereby mediating the degradation of MAVS via the autolysosomal pathway [30]; lumpy skin disease virus (LSDV) ORF142 also degrades STING by recruiting NBR1 and relying on the autophagy pathway [74], which reflects the convergent evolution of immune evasion strategies across different viruses.

Viruses degrade PRRs or their key adaptor molecules through selective autophagy, blocking the production of interferon signals at the source. Degradation of MDA5/RIG-I-like receptor pathway components: The MGF-360-4 L protein of ASFV can act as a “molecular bridge”, recruiting the selective autophagy receptor SQSTM1/p62 to MDA5, mediating its autophagic degradation and thereby inhibiting viral RNA recognition [75]. The nonstructural protein NSP2 of PRRSV enhances the K63-linked ubiquitination of RIG-I, promotes the autophagic degradation of the adaptor protein SH3KBP1, and disrupts downstream signal transduction [76]. Degradation of MAVS signal complexes: MAVS localized in mitochondria is the core hub of the RLR signaling pathway. PRV upregulates the E3 ligase TRIM26, which acts as a scaffold protein to bridge MAVS and the autophagy receptor NDP52, thereby targeting MAVS for autophagic degradation [31]. The PB1 protein of IAV recruits the E3 ligase RNF5 to catalyze the K27-linked polyubiquitination of MAVS; this modification is recognized as a “degradation tag” by the selective autophagy receptor NBR1, leading to MAVS clearance [30]. Despite belonging to different viral species, these viruses have independently evolved similar strategies to target the critical nodes of this pathway. Degradation of the cGAS-STING signaling pathway: Targeting the cytoplasmic DNA-sensing pathway, the ORF142 protein of LSDV directly interacts with STING and recruits NBR1, guiding STING into the autophagic degradation pathway and inhibiting IRF3 activation and type I interferon (IFN-I) production [74].

Even after interferons are produced, viruses can still weaken the antiviral state by degrading key molecules in interferon signal transduction pathways via autophagy. Degradation of JAK-STAT pathway components: The pB125R protein of ASFV directly binds to the type I interferon receptor subunit IFNAR2 and promotes its autophagic degradation, resulting in blocked nuclear translocation of the downstream ISGF3 complex and significantly inhibited expression of interferon-stimulated genes (ISGs) [77]. Degradation of transcriptional regulatory factors: The virulence factor NSs of SFTSV not only retains SAFA (a nuclear viral RNA sensor) in the cytoplasm but also mediates its degradation via SQSTM1/p62-mediated selective autophagy, thereby blocking activation of SAFA-dependent antiviral enhancers [78,79]. CSFV uses its HDAC3 protein to deacetylate and degrade PHGDH, a key enzyme in serine metabolism, indirectly impairing mitochondrial function and inhibiting the MAVS-IRF3 pathway; this process also involves recruitment of p62 and NDP52 [80]. Degradation of kinases and regulatory proteins: The A137R protein of ASFV uses its RING domain to catalyze K63-linked ubiquitination of TBK1 and relies on the ATG5-ATG12-ATG16L1 complex to direct activated TBK1 to lysosomal degradation, directly abrogating the upstream signal for IRF3 phosphorylation [81]. The E protein of PRRSV induces nucleocytoplasmic redistribution of the DDX10 helicase and degrades it through SQSTM1/p62-dependent autophagy, weakening its ability to promote IFN production [82].

Viruses can also regulate autophagy in immune cells (e.g., dendritic cells) to affect their maturation and antigen-presenting functions, achieving cellular-level immune evasion. For example, after infecting plasmacytoid dendritic cells (pDCs), Epstein–Barr virus (EBV) exerts contradictory dual regulation: on the one hand, it triggers massive production of IFN-α via TLR9/7; on the other hand, it blocks TNF-α expression and impairs cellular maturation by interfering with autophagic flux, preventing pDCs from effectively activating T cells and thus establishing persistent infection [83,84,85]. This reflects the sophisticated strategy by which viruses fine-tune the “autophagy-immunity balance threshold” in host cells to achieve immune evasion.

In conclusion, the core mechanism by which viruses utilize autophagy for immune evasion involves precise recognition of key signaling molecules in the type I interferon pathway (e.g., MDA5, MAVS, STING, TBK1, and IRF3) and hijacking of selective autophagy mechanisms by viral proteins (e.g., recruiting receptors such as p62, NBR1, and NDP52) to label them “degradation cargo”. This strategy is characterized by high convergent evolution. For example, IAV and PRV use different viral proteins and different autophagy receptors to achieve the same goal—MAVS clearance. This reflects the convergent evolution of viruses in immune evasion: despite differences in molecular tools, the functional outcome is highly consistent, i.e., suppressing the interferon signaling pathway via autophagy. In-depth analysis of the interaction interfaces between these viral proteins, autophagy receptors, and immune components will provide precise targets for the development of broad-spectrum antiviral drugs.

**Table 5:** Summary of virus-mediated immune evasion via autophagy.

Targeted Immune Pathways/Molecules	Virus/Viral Protein	Autophagy Receptor Used	Brief Overview of Core Mechanism	References
MDA5	ASFV MGF-360-4 L	p62/SQSTM1	The viral protein acts as a bridge, recruiting p62 to MDA5 and mediating its autophagic degradation.	[75]
RIG-I	PRRSV NSP2	SH3KBP1 (Indirect)	Enhance RIG-I ubiquitination, promote the autophagic degradation of the adaptor protein SH3KBP1, and disrupt signaling.	[76]
MAVS	IAV PB1	NBR1	E3 ligase is recruited to catalyze K27 ubiquitination of MAVS, which is recognized by NBR1 and mediates its autophagic degradation.	[30]
	PRV (Through TRIM26)	NDP52	Upregulate TRIM26, bridge MAVS and NDP52, and target MAVS for autophagic degradation.	[31]
STING	LSDV ORF142	NBR1	The viral protein directly interacts with STING and recruits NBR1, guiding the autophagic degradation of STING.	[74]
IFNAR2	ASFV pB125R	Unkonw	The viral protein directly binds to IFNAR2, promoting its autophagic degradation and blocking downstream signaling.	[77]
SAFA (Viral RNA sensor)	SFTSV NSs	p62/SQSTM1	Retain nuclear SAFA in the cytoplasm and degrade it through p62-mediated selective autophagy.	[78,79]
PHGDH (Serine metabolism enzyme)	CSFV HDAC3	p62, NDP52	Deacetylates and degrades PHGDH, affects mitochondrial function, and indirectly inhibits the MAVS-IRF3 pathway.	[80]
TBK1	ASFV A137R	Atg5-12-16L1 complex	It catalyzes K63-linked ubiquitination of TBK1 and directs it to lysosomal degradation via the autophagy complex.	[81]
DDX10 (helicase)	PRRSV E protein	p62/SQSTM1	Induce the nucleocytoplasmic redistribution of DDX10 and degrade it through p62-dependent autophagy.	[82]
pDCs	EBV	TLR9/7, TNF-α	Affect the maturation of dendritic cells	[83,84,85]

### 3.6. Interaction Between Enteroviruses and Autophagy

Enteric viruses exhibit sophisticated strategies to hijack the host autophagy machinery at multiple stages of their life cycle, including replication and release. The core strategy entails repurposing the autophagy pathway, particularly its membrane structures, as key platforms that support viral replication.

During viral replication and assembly, double-membrane vesicles (DMVs) derived from autophagosomes offer an ideal physically sequestered environment and essential lipid components for enteroviral RNA synthesis of enteric viruses, serving as core replication organelles (Figure 5). Multiple enteric viruses harness autophagy to enhance the production and maturation of virions. For example, during coxsackievirus B3 (CVB3) infection, autophagosome production (mCherry-GFP-LC3 positive) can be observed, but the fusion of autophagosomes with late endosomes or lysosomes markedly impairs viral replication [86]. Further in-depth studies have shown that CVB3, through the catalytic activity of its protease 3C, specifically targets and cleaves SNAP29, which then binds to the adaptor protein PLEKHM1. This disrupts the SNARE complex, inhibits autophagic flux, and results in the formation of autophagosome aggregates, which likely serve as replication factories to enhance virus replication [87]. Similarly, PV maturation depends on autophagic factors—inhibiting autophagosome acidification impairs virion maturation [88], and cryo-electron microscopy (cryo-EM) observations reveal that PVs assemble directly on replication membranes, a process dependent on the class III phosphatidylinositol 3-kinase VPS34 [89].

During the viral release process, enteroviruses lack a lipid envelope and thus cannot rely on traditional membrane fusion for egress (Figure 5). To overcome this constraint, they extensively hijack autophagy-associated membrane trafficking pathways to achieve nonlytic release. Enteroviruses remodel autophagosomal membranes to form DMVs and exploit the fusion of the DMVs’ outer membrane with the plasma membrane to release viral particles enclosed within the inner membrane into the extracellular space. This process is independent of traditional SNARE proteins (e.g., syntaxin17) [90]. Studies have demonstrated that nonenveloped viruses also widely interact extensively with the autophagic system; for example, enteroviral particles and their precursors colocalize with LC3-positive autophagosomal membrane structures, with their replication and release depend on autophagy-associated factors [91,92]. Furthermore, the 3C protease of enterovirus D68 can cleave the C-terminal HR2 domain of the mitochondrial fusion protein mitofusin-2, inducing mitochondrial fragmentation and mitophagic degradation, which in turn promotes nonlytic viral release from cells [93].

In summary, enteroviruses universally adopt a strategy of “modulating rather than disrupting” strategy toward autophagy. They do not only inhibit autophagy, but also actively induce autophagosome formation while simultaneously and precisely blocking its degradative function. Ultimately, these viruses successfully reprogram the host’s defensive cellular autophagy system into a favorable tool that supports their key processes: genome release, replication factory establishment, and nonlytic viral release. In-depth elucidation of these mechanisms is crucial for developing novel antiviral strategies targeting such viruses.

## 4. Summary and Outlook

The interaction between autophagy and viral infection represents a complex dynamic interplay. This article systematically delineates the core molecular mechanisms of autophagy (initiation, nucleation, elongation, and fusion) and its regulatory network (centered on mTORC1 and AMPK) and elaborates on the specific ways in which different viruses (e.g., SARS-CoV-2, IAV and HIV) utilize autophagy, including targeted interference with selective autophagy. This facilitates a comprehensive understanding of virus-host interactions at the molecular level.

Antiviral therapy based on autophagy regulation provides a novel direction for clinical intervention. Exploring the interaction interface between viral and autophagic proteins to explore their potential as antiviral targets has garnered extensive attention. For example, coronavirus NSP5 can bind to the autophagy receptor NBR1, cleave NBR1, and disrupt NBR1-mediated degradation of antiviral proteins. Since NSP5 is highly conserved among coronaviruses, blocking the NSP5-NBR1 interaction may confer broad-spectrum antiviral activity [94]. Additionally, natural compounds can exert antiviral effects by regulating autophagy (e.g., enhancing the host’s utilization of autophagy to degrade pathogens) [95,96,97]. HIF-1α can mitigate Japanese encephalitis virus (JEV) infection in neurons by reversing the autophagy inhibition caused by JEV infection [98]. Adamantane derivatives (amantadine and rimantadine) can partially enhance autophagy and suppress the replication of HAV [99].

Despite the substantial potential of targeting virus-autophagy interaction interfaces, their clinical application still faces two challenges. First, autophagy exhibits functional heterogeneity across different cell types; for example, excessive autophagy in neurons can induce neurodegeneration [100]. Second, rapid viral mutation may lead to target escape. Future research should prioritize spatiotemporally specific regulatory strategies. For example, utilizing infection microenvironment-responsive nanotechnology for delivering autophagy agonists/inhibitors allows for precise drug release within infected cells, thereby maximizing antiviral efficacy while minimizing off-target effects on uninfected cells. Additionally, developing bispecific or multispecific molecules capable of simultaneously binding both viral proteins (e.g., coronavirus NSP5) and host autophagy receptors (e.g., NBR1) could specifically interfere with viral immune evasion mechanisms by stabilizing or disrupting key complexes, thereby reducing the risk of drug resistance. Moreover, regulators that target specific selective autophagy pathways (e.g., mitophagy or ER-phagy) instead of global autophagy hold promising precision and safety profiles. Furthermore, it is essential to further elucidate the specific mechanisms of autophagy in viral infection and clarify the differences in the molecular targets through which different viruses hijack autophagy, balance the safety of autophagy regulation to avoid disrupting cellular homeostasis due to overactivation or inhibition of autophagy, develop virus-specific autophagy modulators via high-throughput screening technology, or combine them with immunotherapy to enhance host defense capacity. These endeavors will not only deepen the understanding of virus-host coevolution but also provide theoretical support and practical guidance for the design of novel antiviral strategies.

## Figures and Tables

**Table 1:** Autophagy-related Regulatory Mechanisms.

Phase/Pathway	Core Molecule/Complex	Main Functions	References
Core process			
Start	ULK1 complex (ULK1/2, Atg13, FIP200, Atg101)	Responds to upstream signals (e.g., mTORC1 inhibition) to initiate autophagy.	[7]
Nucleation	PI3K III complex (Beclin1-Vps34-Vps15-ATG14)	Generates PI3P, recruits downstream effector proteins, and forms a phagosome.	[8,9,10]
Extend	Atg12-Atg5-Atg16L1 complex	The Atg12-Atg5-Atg16L1 complex promotes LC3 lipidation as an E3-like enzyme	[11,12,13,14,15,16,17]
	LC3 family	LC3-I is lipidated to LC3-II and anchored to the membrane, driving the elongation and closure of the autophagosome membrane.	
Fusion and Degradation	SNARE complex (e.g., Syntaxin17, SNAP29, VAMP8) and HOPS complex	Mediates the fusion of autophagosomes with lysosomes to form autolysosomes for degrading their contents.	[18,19,20]
Main regulatory pathways			
Nutrition/Energy Sensing	mTORC1	mTORC1: Inhibits autophagy when nutrients are abundant (phosphorylates ULK1).	[21,22,23]
	AMPK	AMPK: Activates autophagy during energy deprivation (activates ULK1 and inhibits mTORC1).	
Growth factor signaling	PI3K-Akt pathway	Activate mTORC1, thereby inhibiting autophagy.	[24]
Stress and bidirectional regulation	p53	Nuclear p53: promotes autophagy. Cytoplasmic p53: inhibits autophagy.	[25]
Epigenetic regulation	Histone deacetylase (e.g., LSD1)	Negatively regulates autophagy through the mTOR pathway.	[21]

## Data Availability

No new data were created or analyzed in this study. Data sharing is not applicable to this article.
